# Claudin-4 Stabilizes the Genome via Nuclear and Cell-Cycle Remodeling to Support Ovarian Cancer Cell Survival

**DOI:** 10.1158/2767-9764.CRC-24-0558

**Published:** 2025-01-07

**Authors:** Fabian R. Villagomez, Julie Lang, Daniel Nunez-Avellaneda, Kian Behbakht, Hannah L. Dimmick, Patricia G. Webb, Kenneth P. Nephew, Margaret Neville, Elizabeth R. Woodruff, Benjamin G. Bitler

**Affiliations:** 1 Division of Reproductive Sciences, Department of Obstetrics and Gynecology, School of Medicine, University of Colorado, Anschutz Medical Campus, Aurora, Colorado.; 2 Department of Immunology and Microbiology, University of Colorado Anschutz Medical Campus, Aurora, Colorado.; 3 Deputy Directorate of Technological Development, Linkage, and Innovation, National Council of Humanities, Sciences, and Technologies, Mexico City, Mexico.; 4 Division of Gynecologic Oncology, Department of Obstetrics and Gynecology, The University of Colorado Anschutz Medical Campus, Aurora, Colorado.; 5 Medical Sciences, Indiana University School of Medicine, Bloomington, Indiana.; 6 Melvin and Bren Simon Comprehensive Cancer Center, Indiana University, Indianapolis, Indiana.; 7 Department of Anatomy, Cell Biology and Physiology, Indiana University, Indianapolis, Indiana.

## Abstract

Alterations in the interplay between the nucleus and the cell cycle during cancer development lead to a state of genomic instability, often accompanied by observable morphologic aberrations. Tumor cells can regulate these aberrations to evade cell death, either by preventing or eliminating genomic instability. In epithelial ovarian cancer, overexpression of claudin-4 significantly contributes to therapy resistance through mechanisms associated with genomic instability regulation. However, the molecular mechanisms underlying claudin-4 overexpression in epithelial ovarian cancer remain poorly understood. In this study, we modified claudin-4 expression and employed a unique claudin mimic peptide to investigate claudin-4’s function. Our findings show that claudin-4 supports ovarian cancer cell survival by stabilizing the genome through nuclear and cell-cycle remodeling. Specifically, claudin-4 induced nuclear constriction by excluding lamin B1 and promoting perinuclear F-actin accumulation, thereby altering nuclear structure and dynamics. Similarly, cell-cycle modifications due to claudin-4 overexpression resulted in fewer cells entering the S-phase and reduced genomic instability in tumors. Importantly, disrupting claudin-4’s biological effects using claudin mimic peptide and forskolin increased the efficacy of PARP inhibitor treatment, correlating with alterations in the oxidative stress response. Our data indicate that claudin-4 protects tumor genome integrity by modulating the crosstalk between the nucleus and the cell cycle, leading to resistance to genomic instability formation and the effects of genomic instability–inducing agents.

**Significance:**

High-grade serous ovarian carcinoma is marked by chromosomal instability, which can serve to promote disease progression and allow cancer to evade therapeutic insults. The report highlights the role of claudin-4 in regulating genomic instability and proposes a novel therapeutic approach to exploit claudin-4–mediated regulation.

## Introduction

Cell-cycle dysregulation is a fundamental hallmark of cancer that drives both altered cell proliferation and genomic instability, making it a critical therapeutic target (1). The cell cycle plays a vital role in nuclear physiology, precisely coordinating nuclear envelope and cytoskeleton dynamics. This regulation ensures proper nuclear remodeling and safeguards genomic integrity throughout each phase of cell progression (2–6). The nucleus is physically connected to cell-to-cell junctions, which play a major role in the interplay between the cell cycle and nuclear physiology. This interplay occurs through the nuclear envelope, a membrane network composed of lamin proteins and surrounded by the linkers of the nucleoskeleton to the cytoskeleton complex, which binds to the cytoskeleton via actin, microtubules, and intermediate filaments (3, 6–11). Mechanistically, the connection between the nucleus and cell-to-cell junctions regulates both morphology and cell-cycle progression through mechanotransduction, regulation of nuclear positioning and shape, spatial organization of tissues, and modulation of cell-cycle checkpoints (2, 12–14). Consequently, significant alterations in nuclear morphology, which are closely linked to genome instability, are commonly observed in cancer (15, 16). Nevertheless, it is known that tumor cells can regulate genomic instability to maintain optimal tumor growth, either by preventing or eliminating this hallmark of cancer (17–19).

Claudin-4 is aberrantly overexpressed in most epithelial ovarian carcinomas (EOC; refs. 19–23) and is associated with resistance to therapy (22, 24) and poor patient survival. This phenomenon is closely related to the regulation of genomic instability.(19, 22) Claudin-4 is a multifunctional protein that has been involved in many cellular functions, including cell proliferation (25) and DNA damage repair (22), but it has been traditionally described as a cell-to-cell junction protein (26, 27). We recently reported that this protein forms a functional axis with the amino acid transporters SLC1A5 and LAT1, playing a crucial role in controlling micronuclei, markers of genomic instability, through autophagy (19). This indicates that claudin-4 actively participates in mitigating genomic instability after it arises (19, 22). Additionally, several studies underscore the potential clinical significance of claudin-4 in the treatment and prognosis of ovarian cancer (19, 20, 22, 26, 28). However, the precise molecular mechanisms by which claudin-4 regulates genomic instability remain largely unexplored.

In this study, we aimed to determine the influence of claudin-4 on cell-cycle progression and nuclear architecture, and how its regulation may lead to changes in genomic instability and therapy resistance in ovarian cancer cells. We used a claudin mimic peptide (CMP) to target a conserved sequence in claudin-4 (19, 20, 29) to disrupt its interactions with partner proteins and induce mis-localization (19, 20, 29, 30). We modulated claudin-4 expression in various EOC cells *in vitro*, including its overexpression in OVCAR8 cells and downregulation in OVCAR3 and OVCA429 cells. We identified a previously unknown “dual regulatory role” of claudin-4 that reshapes the nuclear structure and cell-cycle progression, contributing to the crosstalk between nuclear physiology and the cell cycle. Furthermore, this dual role of claudin-4 was associated with ovarian tumor cell resistance to genome instability formation and cell death by a known genomic instability-inducing agent, such as the PARP inhibitor olaparib (31, 32).

## Materials and Methods

### Cell lines

Human-derived cells, OVCA429 (RRID: CVCL_3936), OVCAR3 (RRID: CVCL_DH37), and OVCAR8 (RRID: CVCL_1629), collected from the Gynecologic Tissue and Fluid Bank, were cultured in RPMI-1640 medium (Gibco, Thermo Fisher Scientific, Cat. #11875) plus 10% heat-inactivated FBS (Phoenix Scientific, Cat. # PS-100, Lot. # 20055-01-01) and 1% penicillin/streptomycin (Corning, Cat. #30-002-CI) at 37°C and 5% CO_2_. HEK293FT (RRID: CVCL_6911) were cultured similarly but in DMEM medium (Gibco, Thermo Fisher Scientific, Cat. #11995040). Cells were only cultured for up to 20 passages and were tested for *Mycoplasma* during the time of experiments (last tested on September 04, 2024) using the *Mycoplasma* PCR Detection Kit (Sigma-Aldrich, cat. # MP0035).

### Inhibition of claudin-4 expression by CRISPRi

Stable transfectants of OVCA429 and OVCAR3-dCas9 cells were generated by co-transfection with the dCas9 vector (pB-CAGGS-dCas9-KRAB-MeCP2, RRID: Addgene_110824) and the Super PiggyBac Transposase Expression Vector (pCRISPRia-v2; RRID: Addgene_84832) at a DNA ratio of 2.5:1, respectively, using Lipofectamine 2000 (Thermo Fisher Scientific, cat: 11668-019) according to the manufacturer’s instructions. Selection was carried out by antibiotic resistance (blasticidin). A guide RNA (gRNA; forward: GCT​GGC​TTG​CGC​ATC​AGG​AC; reverse: GTC​CTG​ATG​CGC​AAG​CCA​GC) specific for human *CLDN4* (claudin-4) was generated in the Broad Institute portal (https://portals.broadinstitute.org/gppx/crispick/public). Subsequently, the pCRISPRia-v2_base (TagBFP) vector was digested (BstXI and BlpI) to insert the gRNA by ligation, followed by cloning using *Escherichia coli* (Stbl3, Thermo Fisher Scientific, cat: C737303). dCas9-expressing cells were transfected with the pCRISPRia-v2_base vector containing gRNA, as indicated above.

### Vectors, lentivirus production, and transduction

HEK293FT cells were transfected using lipocomplexes (Lipofectamine 2000, Thermo Fisher Scientific, cat: 11668-019) containing the viral packaging system of second generation (psPAX2, RRID: Addgene_12260; pMD2.G, RRID: Addgene_12259) as well as the lentiviral construct of interest (pLenti-Lifeact-tdTomato, RRID: Addgene_64048; pLenti-HIFR, RRID: Addgene_192946; and GFP-tagged claudin-4 pLenti-C-mGFP vector; Cat. # RC200490L2, OriGene. From this plasmid, the GFP tag was removed using conventional molecular cloning techniques), respectively. Supernatant from transfected HEK293FT cells was collected, filtered (0.45 µm), used, or stored (−80°C). Also, GFP-tubulin (EGFP-Tubulin-6, RRID: Addgene_56450) was cloned to the pCDH-CMV-MCS-EF1-Puro (EGFP-Tubulin- pCDH-CMV-MCS-EF1-Puro) vector using common cloning techniques (enzymatic restriction, NheI/BamHI; ligation, plasmid sequencing was used to validate our construct) and viral particles were generated as indicated above.

### Cell-cycle analysis by flow cytometry

A total of 2 × 10^5^ cells were seeded onto six-well plates (2 mL RPMI complete medium). The next day, cells were washed (sterile PBS 1×) and the medium was changed to RPMI complete medium (2 mL). After 24, 48, and 96 hours of incubation, cells were washed (PBS 1×), detached (trypsin 0.25 mmol/L), and centrifuged (1,500 rpm/5 minutes). Cell pellets were resuspended in cold PBS 1× and centrifuged (1,500 rpm/5 minutes). Then, PBS was discarded, and cells were fixed using cold ethanol 70% (ethanol, milliQ water, v/v) for 30 minutes at 4°C, then centrifuged (1,500 rpm/5 minutes/4°C). Afterward, cells were washed twice with cold PBS 1×, and the PBS was discarded after centrifugation (1,500 rpm/5 minutes/4°C). Cells were treated with 50 µL of RNAse A (at 100 µg/mL concentration) for 30 minutes at room temperature and then stained with 300 µL of propidium iodide (at 50 µg/mL concentration). Analysis was carried out in the Cancer Center Flow Cytometry Shared Resource (RRID: SCR_022035), University of Colorado Anschutz Medical Campus.

### Colony formation assay

A total of 3 × 10^4^ ovarian tumor cells were seeded onto 24-well plates (1 mL RPMI complete medium), and the next day, cells were washed with PBS. Individual treatment (olaparib) or combination was applied [combination, combo: forskolin (FSK), 5 µmol/L; CMP, 400 µmol/L; and olaparib, 600 nmol/L] in 2 mL RPMI complete medium. Ovarian tumor cells were allowed to grow in the presence of individual or combination treatment for 7 days. OVCA429 cells received one dose of individual treatment due to greater resistance to olaparib (at day 0; olaparib concentration: 120–30,000 nmol/L), and OVCAR3/OVCAR8 cells received two doses of individual treatment due to less resistance to olaparib (at day 0 and day 4; olaparib concentration: 120–1,920 nmol/L). After 7 days of treatment, cells were washed with PBS and fixed (PBS containing 10% acetic acid and 10% methanol) for 10 minutes and stained (using PBS 1× with 0.4% crystal violet and 20% ethanol for 10 minutes). To estimate the number of surviving cells, the cells were destained with PBS containing 10% acetic acid and 10% methanol, and absorbance was read at 570 nm.

### Immunoblot and CMP synthesis

To analyze the protein expression levels of lamin B1, lamin A/C, LAT1, and hypoxia-inducible factor-1α (HIF-1α), tumor cells were scraped from culture plates in the presence of lysis buffer (30 mmol/L Tris HCl pH7.4, 150 mmol/L NaCl, 1% Triton X-100, 10% glycerol, 2 mmol/L EDTA, 0.57 mmol/L phenylmethylsulfonylfluoride, and 1× cOmplete Protease Inhibitor Cocktail), placed on a shaker for 10 minutes and spun at 13,000 rpm for 10 minutes. Protein was separated by SDS-PAGE and transferred to the polyvinylidene difluoride membrane using the TransBlot Turbo system (Bio-Rad). Membranes were blocked with Intercept Blocking Buffer (LI-COR, #927-60001) for 2 hours at room temperature. The following primary antibodies were used and incubated overnight at 4°C: mouse anti-human claudin-4 (Thermo Fisher Scientific, Cat. # 32-9400, RRID: AB_2533096, 1:500 dil), rabbit anti-lamin B1 (Proteintech, Cat. # 12987-1-AP, RRID: AB_2136290, 1:3,600), mouse anti-lamin A/C (Cell Signaling Technology, Cat. # 4777, RRID: AB_10545756, 1: 1,000), rabbit anti-LAT1 (Cell Signaling Technology, Cat. # 5347, RRID: AB_10695104, 1: 1,000), rabbit anti–HIF-1α (Proteintech, Cat. # 20960-1-AP, RRID: AB_10732601; 1: 1,000), rabbit anti-GAPDH (Sigma, Cat. # HPA040067, RRID: AB_10965903, 1: 1,000), and mouse anti–β-actin (Abcam, Cat. # ab8226, RRID: AB_306371, 1: 5,000). Membranes were washed three times for 5 minutes each in TBST (50 mmol/L Tris pH 7.5, room temperature, followed by secondary antibodies (IRDye 680RD Goat anti-Rabbit IgG; RRID: AB_10956166; dilution, 1:20,000; IRDye 800CW Goat anti-Mouse IgG; RRID: AB_621842; dilution, 1:20,000) for 2 hours at room temperature. Membranes were washed again five times for 5 minutes each in TBST. For fluorescent detection, bands were visualized using the LI-COR Odyssey Imaging System. CMP was synthesized as previously reported (29).

### Immunofluorescence

Cells were fixed with paraformaldehyde at 4% (PBS 1×) for 10 minutes, followed by permeabilization (30 minutes, 0.1% Triton X-100, PBS 1×). Blocking was carried out by 2 hours of incubation with BSA at 5% (PBS 1×, RT, shaking). Primary antibodies (as cited above, lamin B1, 1:800; lamin A/C, 1:100; LAT1, 1:100; HIF-1α, 1:100) were incubated (BSA at 2%, PBS 1×) overnight at 4°C while shaking. Secondary antibodies (Alexa Fluor 546 anti-mouse, Thermo Fisher Scientific, cat: A-11030, at 2 µg/mL; Alexa Fluor 647 anti-rabbit, Thermo Fisher Scientific, cat: A32733, at 2 µg/mL) were incubated 2 hours/shaking at room temperature (BSA at 2%, PBS 1×). Nuclei were stained with 4'6-diamidino-2-phenylindole dihydrochloride (DAPI) at 1 µg/mL (PBS 1×) for 10 minutes. All microscopy acquisition was done in the Neurotechnology Center, University of Colorado Anschutz Medical Campus.

### Live-cell imaging

A total of 2 × 10^5^ cells were seeded onto glass bottom dishes (35 mm, No 1.5; MatTek, Cat. # P35G-1.5-14-C) and cultured in 2 mL RPMI complete medium without phenol red (Thermo Fisher Scientific, Cat. # 11835030). Nuclei were stained using Hoechst 33342 1 µmol/L (Thermo Fisher Scientific, 62249). All microscopy acquisition (FV1000, Olympus) was done in the Neurotechnology Center, University of Colorado Anschutz Medical Campus.

### Measurement of reactive oxygen species

A total of 2 × 10^5^ cells were seeded into six-well plates, and the following day, cells were washed with 1× sterile PBS and treated (combo: olaparib, 600 nmol/L; FSK, 5 µmol/L; and CMP, 400 µmol/L for 2 hours). Tert-butyl hydrogen peroxide was used as a positive control (250 µmol/L, 2 hours; all 2 mL complete RPMI medium). To measure reactive oxygen species (ROS), we used the Dichlorodihydrofluorescein diacetate / 2′,7′-Dichlorodihydrofluorescein diacetate (DCFDA/H2DCFDA)—Cellular ROS Assay Kit (Abcam, cat. # ab113851). Cells were stained for ROS using DCFDA at 10 µmol/L (1 mL RPMI medium final volume) for 40 minutes/37°C. Cells were placed on ice and then analyzed via flow cytometry. All analyses were conducted at the Cancer Center Flow Cytometry Shared Resource, University of Colorado Anschutz Medical Campus.

### Statistical considerations

ImageJ (NIH) and Prism software (v9.0) were used for microscopy and statistical data analysis, respectively. At least three independent experiments were conducted for most experiments. Unpaired *t* tests and Mann–Whitney tests, Kruskal–Wallis test, and one-way ANOVA with Dunn’s or Tukey multiple comparisons test were employed, based on data normality distributions and the number of variables. The level of significance was *P* < 0.05.

### Data availability

The Cancer Genome Atlas (TCGA) data are available on dbGAP accession number, PHS000178. Ovarian serous cystadenocarcinoma (TCGA, PanCancer Atlas) was accessed via cBioPortal on April 14, 2020. All other data will made available by the corresponding author upon request.

## Results

### Claudin-4 enables ovarian tumor cells to modify the entry to and exit from cell-cycle phases

To gain insights into claudin-4’s functional effects in ovarian cancer, we selected various EOC cells (OVCAR8, OVCA429, and OVCAR3). OVCAR8 cells, which do not naturally express claudin-4, were engineered to overexpress it. In contrast, claudin-4 expression was downregulated using CRISPR inhibition in OVCA429 and OVCAR3 cells, both of which naturally express claudin-4 (See Supplementary Fig. S1A and S1B). Additionally, we employed a small peptide called CMP, which specifically targets a conserved sequence in claudin-4, potentially affecting claudin-4’s interactions with other protein partners (See Supplementary Fig. S1C and S1D).

**Figure 1:**
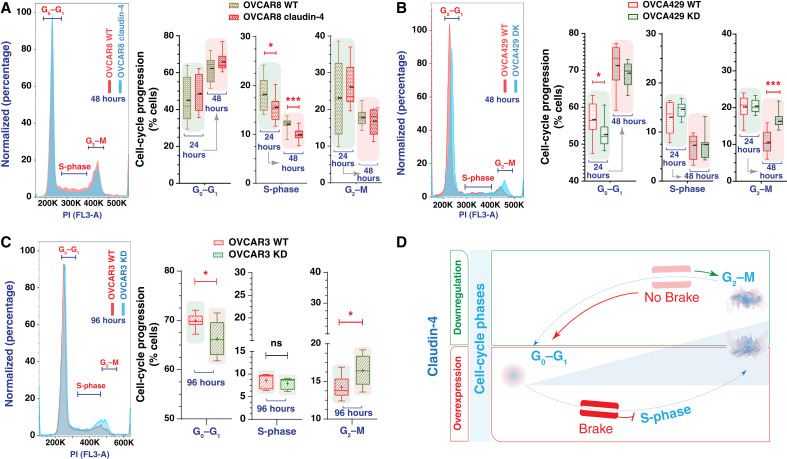
Cell-cycle progression during claudin-4 modulation. Ovarian tumor cells were cultured and stained for PI at 24, 48 (OVCAR8 and OVCA429 cells), and 96 hours (OVCAR3 cells). Subsequently, cell-cycle progression was evaluated via flow cytometry. **A,** Representative histograms of cell-cycle phases during claudin-4 overexpression; right, percentages of cells at each cell-cycle stage. Similarly, the effect of claudin-4 downregulation in OVCA429 (**B**) and OVCAR3 (**C**). **D,** Model illustrating that claudin-4 overexpression reduces the proportion of tumor cells in the S-phase of the cell cycle, whereas its downregulation results in an accumulation of cells in the G2–M phase and a decrease in the G0–G1 phase. (four independent experiments, three independent experiments for OVCAR3 at 96 hours; two-tailed unpaired *t* test; significance *P* < 0.05). Graphs show min to max (**+** indicates mean). KD, knockdown; PI, propidium iodide.

Given the association of claudin-4 with both the cell cycle (28) and genomic instability (19, 22) in ovarian tumor cells, we evaluated cell-cycle progression. These cells were analyzed at different times (post-plating), followed by the evaluation of the cell cycle. Initial assessment of parental cell lines showed that all these cell types could transition through the cell-cycle phases: G0–G1, S-phase, and G2–M. Cells tended to remain in the G0–G1 phase for extended periods, leading to diminished progression into the S and G2–M phases (See Supplementary Fig. S2A) and leading to heterogeneous distributions of cells across the different cell-cycle phases, with an increase in the proportion of cells in the G0–G1 phase over time (See Supplementary Fig. S2B). This behavior may be linked to decreased nutrient availability over time, as suggested by previous studies (19, 33), and evaluation of the cell cycle during starvation conditions (See Supplementary Fig. S2C). Subsequently, we evaluated the effect of claudin-4 overexpression (OVCAR8 claudin-4 cells) and downregulation (OVCA429 and OVCAR3 knockdown cells) on cell-cycle progression. These manipulations led to modifications in the cell-cycle progression compared with wild-type (WT) cells. Claudin-4 overexpression was associated with a significant reduction in cells present in the S-phase (Fig. 1A). At the same time, its downregulation resulted in significantly more cells present in the G2–M and fewer in the G0–G1 phases in OVCA429 and OVCAR3 cells (Fig. 1B and C). However, this phenotype was not significantly observed at the same time points (See Supplementary Fig. S2D), potentially due to differences in doubling time (See Supplementary Fig. S2B). These results align with a previous report noting an increase in the number of cells in the G2–M phase when claudin-4 was downregulated in OVCAR3 cells (28). Overall, our results show that claudin-4 significantly influences the progression of ovarian tumor cells through the cell cycle. Specifically, claudin-4 expression seems to arrest some ovarian tumor cells in the G0–G1 phase, resulting in fewer cells transitioning to the S-phase (Fig. 1A). This finding is further supported by the observation that claudin-4 downregulation in OVCA429 and OVCAR3 cells leads to an increase in the number of cells in the G2–M phase and a decrease in the G0–G1 phase (Fig. 1B and C). These findings suggest that claudin-4 is crucial for precisely controlling cell-cycle phase transitions. Together, these responses suggest that claudin-4 overexpression enhances control over the cell cycle in ovarian cancer cells by slowing progression through the S-phase and ensuring proper entry and exit from the G2–M phase toward the G0–G1 phase (Fig. 1D), potentially mitigating factors that could lead to genomic instability.

### Claudin-4 expression promotes genomic stability in ovarian tumor cells

**Figure 2:**
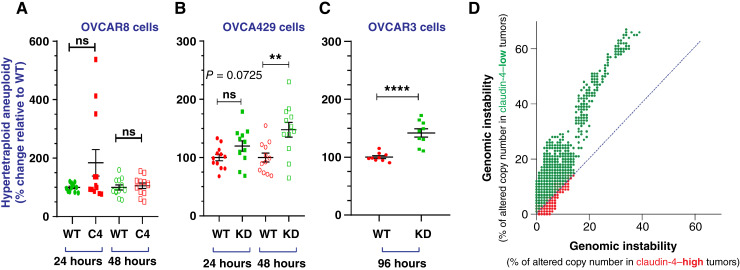
Claudin-4’s association with various forms of genome instability. Ovarian tumor cells were cultured for 24, 48, and 96 hours. Afterward, cells were PI-stained to quantify aneuploidy, a type of genomic instability *in vitro*. Likewise, another type of genomic instability (chromosomic amplifications) was quantified in human tumor samples [TCGA; accession number PHS000178; 2.32% (95% CI, 2.28–2.36) vs. 5.00% (95% CI, 4.93–5.08); *P* < 0.0001]. **A,** Percentages of hypertetraploid (% of variation relative to hypertetraploid observed in WT cells), a form of aneuploidy, during claudin-4 overexpression in OVCAR8 or its downregulation in OVCA429 (**B**) and OVCAR3 cells (**C**), respectively. **D,** Correlation of genomic instability (indicated as % of altered chromosomic copy numbers) in human ovarian tumors associated with levels of claudin-4 expression (four independent experiments, and three independent experiments for OVCAR3 at 96 hours; two-tailed unpaired *t* test; significance *P* < 0.05). Graphs show the mean and SEM. KD, knockdown; PI, propidium iodide.

Genomic amplifications are commonly observed in ovarian cancer (34), and the cell cycle is closely linked to this hallmark of cancer (1). Claudin-4 has been associated with various forms of genomic instability (19, 22). To further explore the association of claudin-4 with genomic instability, we measured hypertetraploid aneuploidy (more chromosomes to those observed in normal G2–M phase of the cell cycle; ref. 35), an indicator of genomic instability, in our cell lines by flow cytometry, as previously reported (See Supplementary Fig. S3A–S3D; ref. 36). We quantified hypertetraploid aneuploidy in EOC cells with claudin-4 overexpression or downregulation during cell-cycle progression. Similar levels were observed in OVCAR8 claudin-4–overexpressing cells compared with WT cells (Fig. 2A), whereas a significant increase in hypertetraploid aneuploidy was found in OVCA429 and OVCAR3 cells with claudin-4 downregulation (Fig. 2B and C). These significant differences suggest chromosomal amplifications resulting from reduced claudin-4 expression. Additionally, the lack of significant changes in genomic instability with claudin-4 overexpression in naturally claudin-4–negative cells (OVCAR8) suggests a potential limiting role of claudin-4 in relation to this form of genomic instability. We analyzed claudin-4 expression in human ovarian tumors using TCGA to support these findings. We categorized tumors as claudin-4–low and claudin-4–High based on quartiles (Q) of mRNA expression, with Q1 representing low and Q4 high (cutoff threshold). We then correlated claudin-4 levels with chromosomal number amplifications to indicate genomic instability. Ovarian tumors with high claudin-4 expression displayed a 2-fold reduction in genomic instability compared with tumors with low claudin-4 expression (Fig. 2D), consistent with previous reports linking claudin-4 expression to reduced genetic mutations (22). As claudin-4 is also implicated in regulating micronuclei via autophagy (19), our results further support claudin-4’s role in regulating genome instability.

### Claudin-4 remodels the nuclear structure by altering the nuclear lamina and actin cytoskeleton

**Figure 3:**
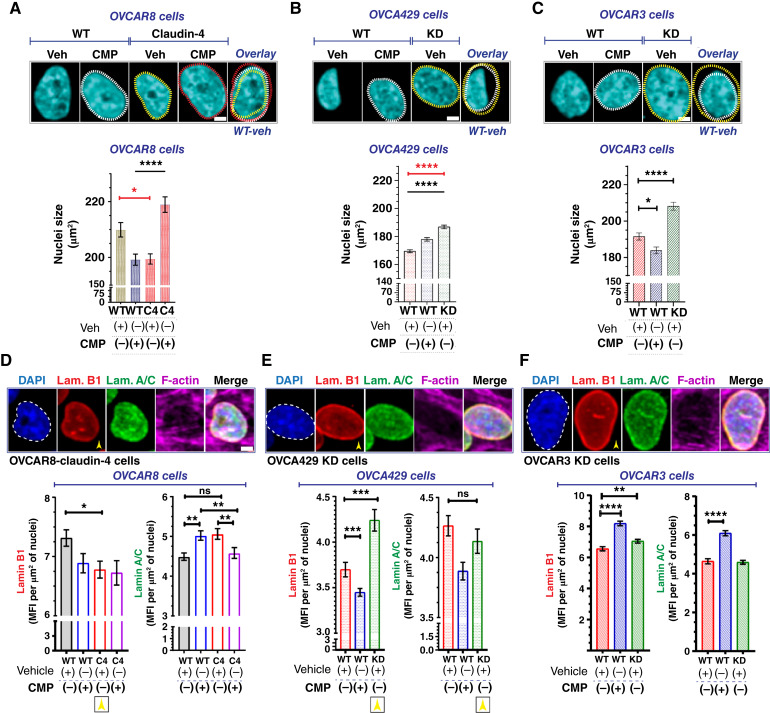
Remodeling of nuclear morphology and the nuclear lamina during claudin-4 disruption. Ovarian tumor cells were treated with CMP (400 µmol/L) for 48 hours, and then cells were stained to mark the nuclear lamina (using antibodies against lamin B1 and lamin A/C) and the nuclei (using DAPI). Subsequently, we performed a morphometric characterization. **A,** Top, confocal images showing (maximum projections) nuclei size of OVCAR8 as well as OVCA429 (**B**) and OVCAR3 cells (**C**). Bottom, corresponding quantification for (**A–C**; *n* = OVCAR8, 1711 cells; OVCA429, 2,630 cells; OVCAR3, 2,365 cells; two-tailed Mann–Whitney test, Kruskal–Wallis test with Dunn’s multiple comparisons; three independent experiments). **D,** Top, selected confocal images (maximum projections) showing nuclear lamina components corresponding to OVCAR8 cells overexpressing claudin-4 and claudin-4 downregulation in OVCA429 (**E**) and OVCAR3 cells (**F**). Bottom, corresponding quantification of nuclear accumulation of lamin B1 and lamin A/C (yellow arrowheads highlight comparison of lamin B1 during claudin-4 overexpression and downregulation) under different conditions (three independent experiments; Kruskal–Wallis test with Dunn’s multiple comparisons. (Significance, *P* < 0.05). Graphs show mean and SEM, scale bar, 5 µm. KD, knockdown; MFI, mean fluorescence intensity; Veh, vehicle.

Major changes in nuclear shape are closely tied to genome instability in cancer (15, 16). Also, it has been suggested that nuclear size and chromosomal amplifications are positively correlated in ovarian cancer cells (15, 16, 37–40). To gain further insights into claudin-4’s role in genomic instability, we conducted a morphometric characterization of ovarian tumor cell nuclei during claudin-4 expression modulation and CMP treatment. EOC cells were treated or left untreated, then stained via immunofluorescence to mark the main components of the nuclear lamina (lamin B1 and lamin A/C; ref. 41), with phalloidin to label the actin cytoskeleton, as previously reported (42), and DAPI to mark DNA. Specifically, we found that one of the morphologic indicators evaluated, nuclear size, was reduced in claudin-4–overexpressing cells compared with WT cells, and this phenotype was reversed by CMP treatment (Fig. 3A). Conversely, nuclear size expanded when claudin-4 was downregulated (Fig. 3B and C). However, CMP only increased nuclear size in OVCA429 cells, whereas in OVCAR3 cells, the reverse effect occurred. Although it is clear that claudin-4 plays a role in regulating nuclear size and that CMP can moderate its effects, this finding highlights the intertumoral diversity of ovarian tumor cells, suggesting cell line–specific effects (19, 43).

Furthermore, claudin-4 overexpression led to reduced accumulation of lamin B1 in the nucleus (Fig. 3D), whereas its downregulation significantly increased lamin B1 nuclear localization (Fig. 3E and F). Notably, claudin-4 is known to be localized in the nucleus of ovarian tumor cells (26), where we observed an inverse relationship between claudin-4 expression and lamin B1 nuclear localization (Fig. 3D–F). Given that no significant changes were detected in the expression levels of lamin B1 or lamin A/C (See Supplementary Fig. S2E–S2G), it seems that claudin-4 expression primarily affects the localization of lamin B1 (41). In addition, targeting claudin-4 via CMP treatment affected the nuclear lamina as well, causing significant changes in the nuclear localization of both lamin B1 and lamin A/C in EOC cells (Fig. 3D–F). Specifically, in OVCAR8 WT cells, treatment with CMP led to increased nuclear localization of lamin A/C but not lamin B1. Interestingly, this CMP effect on lamin A/C was reversed in claudin-4 overexpressing cells (Fig. 3D), indicating a specific role of claudin-4 in modulating the CMP-induced changes in lamin A/C distribution. Additionally, in OVCA429 WT cells, which naturally express claudin-4, CMP treatment was associated with a decreased accumulation of lamin B1, and a similar trend in lamin A/C (Fig. 3E). In contrast, we observed increased lamin B1 and lamin A/C levels in OVCAR3 WT cells (Fig. 3F). Thus, the effect of CMP on nuclear lamina components (lamin B1 and lamin A/C) varied among different ovarian cancer cell lines. This variation also suggests cell line–specific effects, potentially associated with differences in the proteins that interact with claudin-4 in these cells, which could influence CMP’s ability to target claudin-4’s role in the nuclear lamina (19, 26, 29).

**Figure 4:**
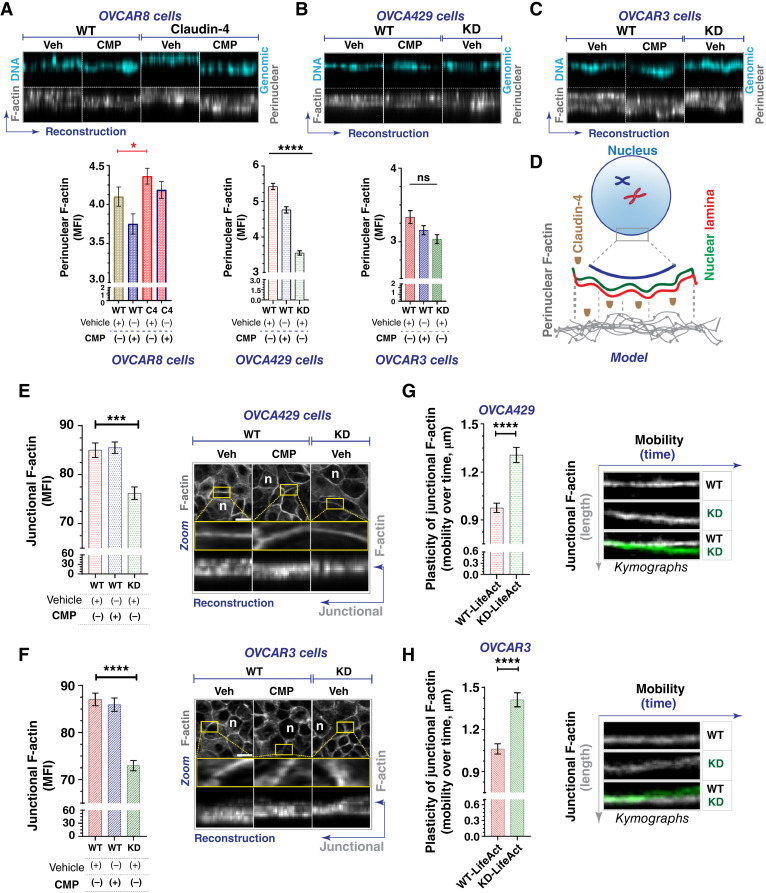
Claudin-4’s effect on the actin cytoskeleton. Ovarian tumor cells were treated with CMP (400 µmol/L) for 48 hours and stained to mark the actin cytoskeleton using phalloidin. In addition, cells were engineered to express LifeAct to mark the actin cytoskeleton in living cells. Afterward, we performed a morphologic and kymograph analysis. **A,** Top, reconstructions of perinuclear F-actin and genomic DNA (from confocal z-stacks) for OVCAR8, OVCA429 (**B**), and OVCAR3 cells (**C**). Bottom, corresponding quantification of perinuclear F-actin for **A–C**, respectively. OVCAR8, 1711 cells; OVCA429, 2630 cells; OVCAR3, 2365 cells; two-tailed Mann–Whitney test, Kruskal–Wallis test with Dunn’s multiple comparisons). **D,** Remodeling effect of claudin-4 in the nuclear architecture, considering both nuclear lamina and perinuclear F-actin. **E** and **F,** left, Quantification of junctional F-actin from reconstructions (from confocal z-stacks). Right, confocal images (maximum projection) and zoom, followed by reconstruction of selected ROIs (at junctional F-actin) from OVCA429 cells (top) and OVCAR3 cells (bottom), respectively (*n* = OVCA429, 783 cells; OVCAR3, 825 cells; Kruskal–Wallis test with Dunn’s multiple comparisons). **G** and **H,** Kymographs illustrating the movement of junctional F-actin (vertical gray arrow) over time (horizontal blue arrow), generated from different ROIs during confocal live-cell imaging of transduced OVCA429 cells (top; *n* = 142) with LifeAct to mark F-actin (without any stimuli and cultured for 24 hours) and OVCAR3 cells (bottom; *n* = 116), respectively (two-tailed Mann–Whitney test; three independent experiments; significance, *P* < 0.05). Graphs show mean and SEM. KD, knockdown; MFI, mean fluorescence intensity; ROI, regions of interest; Veh, vehicle.

Polymeric actin (F-actin) was also affected during claudin-4 modulation, particularly in the perinuclear region and the cytoplasmic fibers (See Supplementary Fig. S4A–S4C). F-actin was more localized in the perinuclear region during claudin-4 overexpression (Fig. 4A) and exhibited the opposite effect during its downregulation, especially in OVCA429 cells (Fig. 4B and C). Additionally, all ovarian tumor cells treated with CMP showed a pattern of reduced perinuclear F-actin localization, suggesting that both expression and proper claudin-4 localization are required for perinuclear F-actin accumulation. Interestingly, we observed a positive correlation between claudin-4 expression levels and perinuclear F-actin accumulation (Fig. 4A–D). This phenotype and the observed inverse relationship between claudin-4 expression and lamin B1 nuclear localization (Fig. 3D–F) suggest a potential competitive exclusion mechanism (44, 45) that could impact lamin B1 dynamics. In addition, we examined F-actin localization in other cellular regions, particularly at cell-to-cell junctions (junctional actin). We detected significantly lower concentrations of F-actin in this region during claudin-4 downregulation in both OVCA429 and OVCAR3 cells (Fig. 4E and F). However, CMP treatment did not affect this accumulation, suggesting that the contribution of claudin-4 to the maintenance of junctional F-actin is less sensitive to CMP’s effects compared with perinuclear F-actin. To better support the role of claudin-4 in F-actin localized at cell-to-cell junctions, we generated ovarian tumor cells expressing LifeAct (a marker of F-actin compatible with living cells). We performed time-lapse confocal imaging and kymograph analysis to capture the temporal motion of junctional F-actin, as previously reported (42, 45). Following claudin-4 downregulation, the cellular connections between ovarian tumor cells displayed increased mobility and progressively became more irregular, suggesting significant alterations in the plasticity of cell-to-cell interactions. (Fig. 4G and H). This observation aligns with the results observed in fixed cells (Fig. 4E and F) and strongly suggests that claudin-4 contributes to one of the characteristics of cell-to-cell plasticity: mobility (46).

Furthermore, it is known that cell-to-cell junctions are physically connected with the nuclear lamina and perinuclear F-actin through the cytoskeleton (6, 47), which influences the positioning of the nucleus for proper cell-cycle progression (6, 47, 48). Because we observed changes in F-actin and microtubule networks in living cells (See Supplementary Fig. S5A and S5B), and given that CMP treatment affected the nuclear lamina and perinuclear F-actin, we hypothesized that the mobility of the nucleus could also be affected by disrupting claudin-4 in living cells. Consequently, we evaluated nuclear mobility during CMP treatment, observing a significant reduction in nuclear mobility compared with untreated cells (See Supplementary Fig. S5C). This effect may result from decreased cytoskeletal connections to the nucleus (3, 6, 47). Significant modifications in nuclear morphology, the nuclear lamina, and the actin cytoskeleton indicate remodeling of the nuclear structure. Thus, claudin-4’s effect on nuclear structure remodeling is feasible (See Supplementary Fig. S5D) and is related to the cell cycle, potentially by altering the dynamics of the nuclear lamina and perinuclear F-actin, which may support genome stability and therapy resistance in ovarian tumor cells.

### Combined CMP and FSK treatment reduces the survival of EOC cells during olaparib therapy

**Figure 5:**
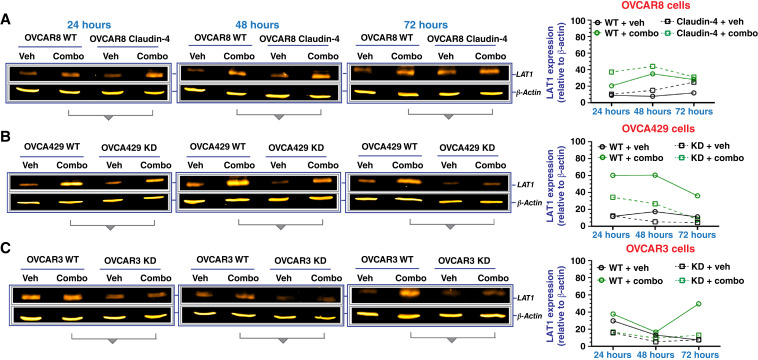
Effect of combining olaparib, FSK, and CMP on LAT1 expression. Ovarian tumor cells were treated with a tripartite combination of olaparib (600 nmol/L), FSK (5 µmol/L), and CMP (400 µmol/L) for different time points. Subsequently, cell lysates were obtained to carry out immunoblotting for LAT1. **A–C** LAT1 protein expression at different time points in OVCAR8, OVCA429, and OVCAR3 cells, respectively. On the right are graphs showing the corresponding quantification of LAT1 from **A–C** relative to loading control. KD, knockdown; Veh/veh, vehicle.

High expression of claudin-4 predicts poor patient survival (See Supplementary Fig. S6A), which is related to the development of therapy resistance, including paclitaxel and the PARP inhibitor, olaparib (22, 28). Consequently, to gain further insight into the potential of targeting claudin-4’s functional effects, such as those observed in cell cycle and nuclear remodeling in ovarian cancer cell survival, we evaluated the effects of CMP and FSK on enhancing olaparib efficacy in a 7-day colony formation assay. FSK is a compound that modifies actin polymerization (49–51) and increases the expression of LAT1 (52), a protein functionally related to claudin-4 (19). FSK also functions to increase levels of cAMP through the activation of adenyl cyclase (53). Critically, FSK has been reported to have anti-ovarian tumor activity by improving the efficacy of a PARP inhibitor (54). We first tested various concentrations of olaparib (120–30,000 nmol/L) on our EOC cells (OVCAR8, OVCA429, and OVCAR3; See Supplementary Fig. S6B and S6C). Given the sensitivity of OVCAR8 and particularly OVCAR3 to higher concentrations, we selected lower olaparib concentrations to assess the potential effects of CMP and FSK. At these selected olaparib concentrations, claudin-4 overexpression in OVCAR8 cells did not increase resistance to olaparib (See Supplementary Fig. S6D). In OVCA429 cells, downregulation of claudin-4 did not enhance olaparib efficacy (See Supplementary Fig. S6E). However, in OVCAR3 cells, claudin-4 downregulation significantly reduced survival at 120 and 240 nmol/L compared with WT cells (See Supplementary Fig. S6F), consistent with the sensitivity observed in the same cells upon claudin-4 downregulation (22). Furthermore, we confirmed that FSK upregulates LAT1 expression (See Supplementary Fig. S6G–S6I), and its anti-ovarian tumor activity alone and combined with CMP (See Supplementary Fig. S6J and S6K). Interestingly, upregulation of LAT1 was also observed when combining olaparib, CMP, and FSK to treat EOC cells at different times (Fig. 5A–C). These results strongly suggest that FSK can increase LAT1 expression, consistent with another report (52), suggesting that the reported link between claudin-4 and LAT1 could be altered due to the effect of FSK (19). Also, we observed increased expression of lamin B1 during FSK treatment in OVCAR8 WT and OVCA429 WT cells [See Supplementary Fig. S6G and S6H (Bottom)], and even though the expression of lamin B1 was not consistent over time and showed more variations during combination treatment (olaparib, CMP, and FSK; See Supplementary Fig. S7A–S7C), its intracellular distribution exhibited clear differences due to our tripartite treatment. For example, this treatment led to the formation of nuclear lamina blebs in OVCAR8 and OVCAR3 cells, whereas in OVCA429 cells, it correlated with a reduced accumulation of cytoplasmic puncta, both of which were enriched with lamin B1 (See Supplementary Fig. S7D–S7I). Thus, it seems that the combination treatment (olaparib, FSK, and CMP) disrupts LAT1 expression and the intracellular distribution of lamin B1.

**Figure 6:**
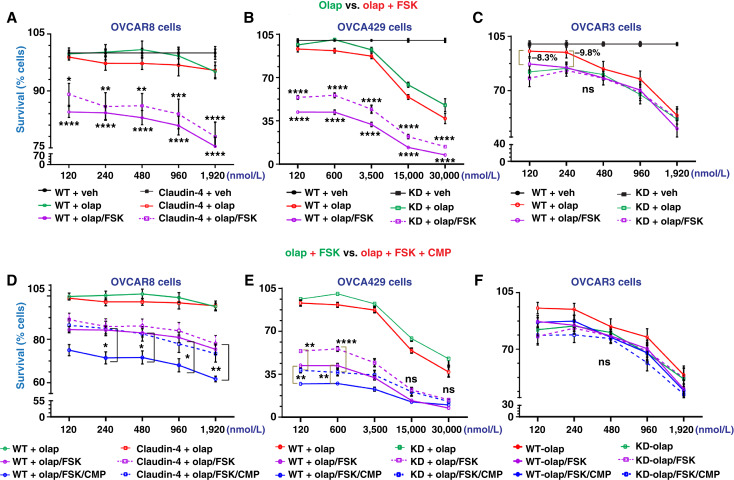
Impact of targeting claudin-4’s functional effects via CMP and FSK on ovarian cancer cell survival. Olaparib treatment was used as a reference (Supplementary Fig. S6D–S6F) to evaluate the effects of CMP and FSK on cell survival using the 7-day colony formation assay and crystal violet staining. Cells were treated as follows: two cycles of treatment (at days 0 and 3) for OVCAR8 and OVCAR3 cells and one treatment for OVCA429 (at day 0). **A,** Percentage of tumor cell survival during olaparib treatment vs. olaparib + FSK (5 µmol/L) and claudin-4 overexpression, and similar information during claudin-4 downregulation in OVCA429 cells (**B**) and OVCAR3 cells (**C**). **D,** Percentage of tumor cell survival during olaparib + FSK (5 µmol/L) vs. olaparib + FSK (5 µmol/L) + CMP (400 µmol/L) and claudin-4 overexpression, and similar information during claudin-4 downregulation in OVCA429 cells (**E**) and OVCAR3 cells (**F**), respectively (three independent experiments; two-way ANOVA; significance *P* < 0.05). Graphs show mean and SEM. KD, knockdown; Olap/olap, olaparib; Veh/veh, vehicle.

In the colony formation assay, the olaparib and FSK combination treatment resulted in a more significant decrease in ovarian cancer cell survival than olaparib only, especially in OVCAR8 and OVCA429 cells (Fig. 6A and B), suggesting that FSK enhances the efficacy of olaparib treatment in these cells. However, this enhancement of olaparib efficacy in reducing cell survival was only increased by 8.3% and 9.8% in OVCAR3 naturally expressing claudin-4 (at 120 and 240 nmol/L of olaparib) compared with olaparib alone, without reaching statistical significance (Fig. 6c). The muted effect of the combined treatment on OVCAR3 cells may be due to the amplification of KRAS, unlike the other ovarian cancer cells (43). Intracellular levels of cAMP are increased by FSK treatment (53), which interacts with MAPK signaling (55). KRAS is a master regulator of MAPK signaling (56) and can lead to sustained MAPK signaling (57, 58), which may oppose the effects of FSK, causing the treatment to be ineffective in OVCAR3 cells.

In OVCAR8 claudin-4–overexpressing cells, olaparib/FSK efficacy was reduced compared with WT cells at all olaparib concentrations tested (Fig. 6A), suggesting that claudin-4 overexpression may enhance resistance to olaparib/FSK therapy. We then performed a triple combination experiment with olaparib, FSK, and CMP in the colony formation assay. Notably, this combination further enhanced the effect of olaparib alone or olaparib with FSK in EOC cells, especially in OVCAR8 and OVCA429 cells (Fig. 6D and E). Specifically, in OVCAR8 claudin-4–overexpressing cells, cell survival was reduced by 12.4% with the triple combination compared with olaparib alone (120 nmol/L). In OVCA429 WT cells (naturally expressing claudin-4), the triple combination resulted in a 65.9% reduction in cell survival compared with olaparib (120 nmol/L) alone. Conversely, in OVCAR3 cells, the triple combination did not further reduce survival compared with olaparib/FSK or olaparib alone (Fig. 6F), with only an 8.7% reduction in survival compared with olaparib (120 nmol/L) alone. Overall, these results suggest that targeting claudin-4’s functional effects through CMP and FSK reduces EOC cell survival during olaparib treatment, highlighting claudin-4’s potential to decrease olaparib resistance and promote ovarian cancer cell death.

### Enhanced oxidative stress response in ovarian tumor cells against combined olaparib, FSK, and CMP treatment

**Figure 7:**
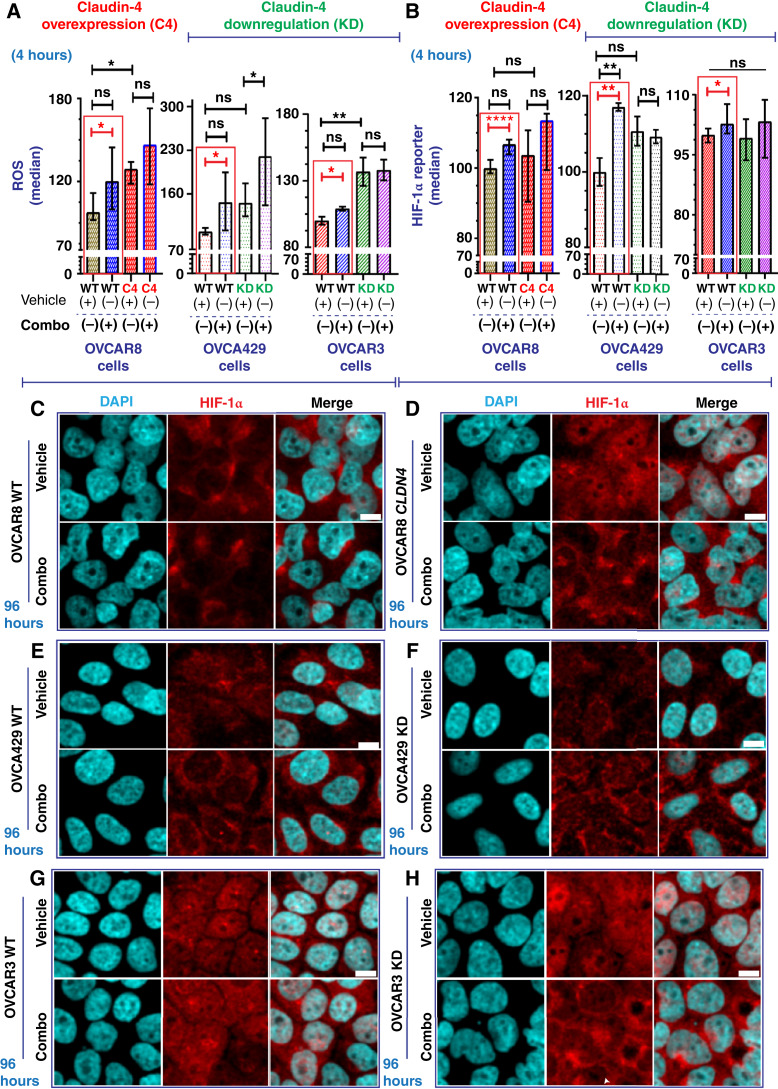
Cellular stress response evaluation in ovarian tumor cells treated with olaparib, FSK, and CMP. Ovarian tumor cells were treated with a tripartite combination of olaparib (600 nmol/L), FSK (5 µmol/L), and CMP (400 µmol/L) for 4 hours to analyze ROS as well as a reporter gene for HIF-1α via flow cytometry. The same cells were treated similarly for 96 hours and then stained using immunofluorescence to mark lamin B1. ROS generation is indicated as normalization relative median of OVCAR8 WT, OVCA429 WT, and OVCAR3 WT cells without treatment (**A**; two independent experiments; unpaired *t* test, red rectangle; one-way ANOVA and Tukey multiple comparison test, *P* < 0.05). Reported HIF-1α is indicated as normalization relative median of OVCAR8 WT, OVCA429 WT, and OVCAR3 WT cells without treatment (**B**; three independent experiments; unpaired *t* test; one-way ANOVA and Tukey multiple comparison test, *P* < 0.05). **C** and **D,** Confocal images showing HIF-1α during claudin-4 (*CLDN4*) overexpression in OVCAR8 cells treated or not as indicated above at 96 hours. **E** and **F,** Confocal images showing HIF-1α during claudin-4 downregulation in OVCA429 cells treated or not as indicated above at 96 hours. **G** and **H,** Confocal images showing HIF-1α during claudin-4 downregulation in OVCAR3 cells treated or not as indicated above at 96 hours. Graphs show the median with 95% CI. Scale bar, 10 µm. CI, confidence interval; KD, knockdown; Veh, vehicle.

In the cells treated with the triple combination, we observed a significant increase in ROS generation and HIF-1α expression (Fig. 7A and B; See Supplementary Fig. S8A–S8C). These findings indicate an upregulation of hypoxia-related elements (59, 60), suggesting an oxidative stress response to counteract the treatment effects (61, 62). Hypoxia, common in tumors and prolonged cell cultures (32, 63, 64), allows tumor cells to adapt to low-oxygen environments (61, 62), contributing to therapy resistance (65, 66). This condition triggers HIF-1α, a key oxygen sensor (59), and ROS production (60), leading to an oxidative stress response (67, 68). HIF-1α and ROS are interrelated in this stress response (69), and tumor cells induce ROS production in response to PARP inhibitors (54, 70). Interestingly, proteins associated with claudin-4’s functional effects, such as lamin B1 (Fig. 3) and LAT1 (19), participate in cellular oxidative stress responses (71–73).

Notably, claudin-4 overexpression correlated with significantly more ROS production [Fig. 7A (left)] but not HIF-1α [Fig. 7B (left)], implying that claudin-4 overexpressing cells may have elevated basal ROS levels, potentially diminishing therapy efficacy (74) due to a protective effect of perinuclear F-actin (Fig. 4A; refs. 75, 76). Supporting this, during claudin-4 downregulation, the positive relationship between claudin-4 overexpression and increased ROS generation was lost in OVCA429 cells [Fig. 7A (middle)]. Unlike OVCAR8 overexpressing claudin-4 [Fig. 7A (left)], the ROS response to treatment was significantly higher in OVCA429 claudin-4 knockdown cells [Fig. 7A (middle)], which also exhibited reduced levels of perinuclear F-actin (Fig. 4B). In contrast, claudin-4 downregulation in OVCAR3 cells led to increased ROS production [Fig. 7A (right)], with no changes in HIF-1α expression [Fig. 7B (right)] and stable perinuclear F-actin (Fig. 4C). Although HIF-1α remained largely unaffected by claudin-4 overexpression or downregulation (Fig. 7B), a significant increase was observed in HIF-1α levels following treatment in OVCA429 WT cells. This suggests potential differences in HIF-1α–mediated oxidative stress response between OVCA429 WT and OVCAR3 WT cells [Fig. 7B (middle and right)]. Variations in ROS production between OVCA429 and OVCAR3 cells may be due to KRAS amplification in OVCAR3 cells (19, 43), which can enhance ROS generation in tumor cells (77). Thus, the effect of our tripartite combination on inducing ROS could be masked by KRAS amplification.

Because claudin-4 modulation did not show significant changes in HIF-1α expression (Figs. 7B, at 4H), we investigated its intracellular distribution using immunofluorescence. We observed notable changes in HIF-1α localization linked to claudin-4 modulation and extended treatment duration. Specifically, claudin-4 overexpression led to increased nuclear accumulation of HIF-1α (Fig. 7C and D) and higher protein levels at later time points (See Supplementary Fig. S8D). The treatment further altered HIF-1α distribution and upregulated its expression in OVCAR8 cells (Fig. 7C and D; See Supplementary Fig. S8D). Claudin-4 downregulation in OVCA429 led to reduced HIF-1α levels (Fig. 7E and F; Supplementary Fig. S8E), whereas in OVCAR3 cells, it resulted in increased HIF-1α levels (Fig. 7G and H; Supplementary Fig. S8F). Notably, claudin-4 downregulation in OVCA429 cells caused HIF-1α to accumulate at the plasma membrane, an effect that was enhanced by treatment (Fig. 7E and F) and partially mirrored in OVCAR3 cells (Fig. 7H). These findings underscore a link between claudin-4 and HIF-1α, indicating that oxygen regulation and related factors, such as ROS (54), could play a role in claudin-4–mediated resistance to olaparib. They also suggest that targeting the oxidative stress response could enhance olaparib efficacy by disrupting claudin-4’s functional effects in ovarian cancer cells, especially OVCAR3 cells.

## Discussion

This study found that claudin-4 protects ovarian cancer cells by remodeling nuclear structure and slowing cell-cycle progression. This mechanism is associated with resistance to genomic instability and to the effects of genomic instability–inducing agents like the PARP inhibitor olaparib. Thus, targeting claudin-4 could reduce the required dosage of olaparib to induce cell death in ovarian cancer cells, potentially decreasing therapy resistance.

Claudin-4 plays a crucial role in regulating the cell cycle, nuclear lamina, and cytoskeleton dynamics. Claudin-4 overexpression resulted in fewer cells in the S-phase, suggesting that this protein may act as a brake, delaying ovarian cancer cells’ entry into the S-phase (Fig. 1D). This phenotype correlated with claudin-4’s impact on nuclear structure and the cytoskeleton, leading to nuclear constriction and suggesting that claudin-4 overexpression could generate mechanical forces that shape the nucleus, preventing its enlargement (3, 47, 78). The nucleus is typically larger in G2–M than that in S or G1–G0 phases (79). We observed a correlation between claudin-4 modulation’s impact on nuclear size and its influence on cell-cycle progression. Claudin-4 overexpression and its downregulation correlated with nuclei constriction and expansion, respectively (Fig. 3A–C), which aligns with a reduced number of cells observed in the S-phase during claudin-4 overexpression (Fig. 1a). Conversely, more cells were noted in the G2–M phase during claudin-4 downregulation (Fig. 1B and C). In addition, it is reported that lamin B1 regulates the entry into the S-phase (80), and our data indicated that claudin-4 modulation affected the nuclear localization of this protein. Specifically, claudin-4 caused the displacement of lamin B1 from the nucleus and promoted the stabilization of F-actin at the perinuclear region (Fig. 4). This phenotype could be linked to a reported exclusion mechanism mediated by fascin and actinin, which compete to bundle F-actin in different actin networks (44, 45). Thus, in our *in vitro* models, more claudin-4 expression leads to a slowed progression through the cell cycle, possibly allowing repair of DNA damage or increased regulation of chromosomal separation (Fig. 2; ref. 22). Overall, these results highlight claudin-4’s role in modulating the interplay between nuclear physiology and cell-cycle progression, which may help reduce the formation of genomic instability, underscoring claudin-4’s potential clinical impact in ovarian tumors (See Supplementary Fig. S6A; ref. 22) through control of the cell cycle and maintenance of genome integrity.

As we reported previously with OVCAR3 cells (22), claudin-4 downregulation in these cells is associated with sensitivity to olaparib (See Supplementary Fig. S6F) but not in OVCA429 cells. These differences underscore the heterogeneity among ovarian cancer cells and emphasize the importance of claudin-4 in modulating the response to PARP inhibitors in high-grade serous ovarian carcinoma, the most prevalent subtype of ovarian cancer, which accounts for more than 75% of all ovarian cancer cases (19, 22, 43). Guidelines to use olaparib are changing according to new information generated (81). However, this treatment is known for inducing catastrophic genomic instability, particularly during mitosis (82), in BRCA-deficient tumors such as ovarian and breast cancer (31, 32, 83), but it induces tumor cell death regardless of such mutations (54, 84–87). We reasoned that claudin-4’s reduction of genomic instability and slowing of mitosis may interfere with olaparib’s effects on OVCAR8, OVCA429, and OVCAR3 cells, which, despite classification discrepancies (OVCAR8 and OVCA429), are recognized as ovarian cancer cell lines (43, 88–98). Therefore, we targeted the claudin-4’s functional effects related to F-actin and LAT1 (19) using CMP and FSK to increase olaparib efficacy. Treatment with CMP and FSK—potentially involving alterations in LAT1 and lamin B1 (Fig. 5A–C; See Supplementary Figs. S6 and S7)—in combination with olaparib, led to greater reductions in survival across all ovarian cancer cells tested at the lowest concentrations of olaparib (Fig. 6). Furthermore, because ovarian cancer cells were cultured for 7 days in the colony formation assay, we speculated that hypoxia may play a role in claudin-4–mediated resistance to olaparib. This hypothesis is supported by the close link between claudin-4 and HIF-1α in regulating hypoxia through a feedback mechanism that may affect autophagy (99) and a similar association between LAT1 and HIF-1α under these conditions (100, 101). Our evaluation of claudin-4’s role in therapy resistance revealed that its effect was associated with previously reported biological interactions, including LAT1 (19), HIF-1α (Fig. 7B–H; 99), and lamin B1, in potential cellular processes such as autophagy, hypoxia, and nuclear lamina remodeling, respectively. This is particularly evident in the evaluation of an oxidative stress response (Fig. 7) during the triple combination treatment. This treatment resulted in a significant increase of ROS and HIF-1α in all ovarian cancer cells (Fig. 7), suggesting that ovarian cancer cells generate an oxidative stress response to counteract olaparib treatment (54, 70), potentially through changes in the metabolism of mitochondria (102). Because an excessive oxidative stress response can lead to cell death (103) and the nuclear lamina can protect against ROS (73), these data suggest that the claudin-4’s role in remodeling the nuclear architecture (Figs. 3 and 4) may help protect tumor cells during excessive oxidative stress response during olaparib treatment. Consequently, modulating the oxidative stress response could further potentiate the combined olaparib/FSK/CMP in reducing ovarian cancer cell survival. For instance, previous studies have shown that FSK and metformin can decrease oxidative stress (104), which could interfere with the response of ovarian tumor cells to the tripartite treatment (Figs. 6 and 7). Additionally, the combination of metformin with olaparib inhibits the proliferation of ovarian cancer cells (105). Our results highlight the potential of this tripartite treatment strategy to reduce therapy resistance in *in vivo* models.

## Supplementary Material

Supplementary Figure 1 Confirmation of claudin-4 modulation via genetic and pharmacologic approaches.

Supplementary Figure 2 Claudin-4 dependent cell cycle and Lamin expression.

Supplementary Figure 3 Gating strategy for hypertetraploid aneuploidy in epithelial ovarian cancer cells.

Supplementary Figure 4 Nuclei morphology following claudin-4 inhibition.

Supplementary Figure 5 Claudin-4 dependent actin reorganization.

Supplementary Figure 6 Claudin-4 modulation and responses to forskilin and olaparib.

Supplementary Figure 7 Lamin B1 expression and nuclei morphology after tripartite treatment.

Supplementary Figure 8 Reactive oxygen species positive control in cell lines.
