# Supplementary material for: Claudin-4 Stabilizes the Genome via Nuclear and Cell-Cycle Remodeling to Support Ovarian Cancer Cell Survival
Source: Cancer Res Commun. 2025 Jan 7;5(1):39–53. doi: 10.1158/2767-9764.CRC-24-0558 (PMC11705808; doi:10.1158/2767-9764.CRC-24-0558)
Supplement: Supplementary Figure 1 — Confirmation of claudin-4 modulation via genetic and pharmacologic approaches. [file crc-24-0558_supplementary_figure_1_suppsf1.docx]

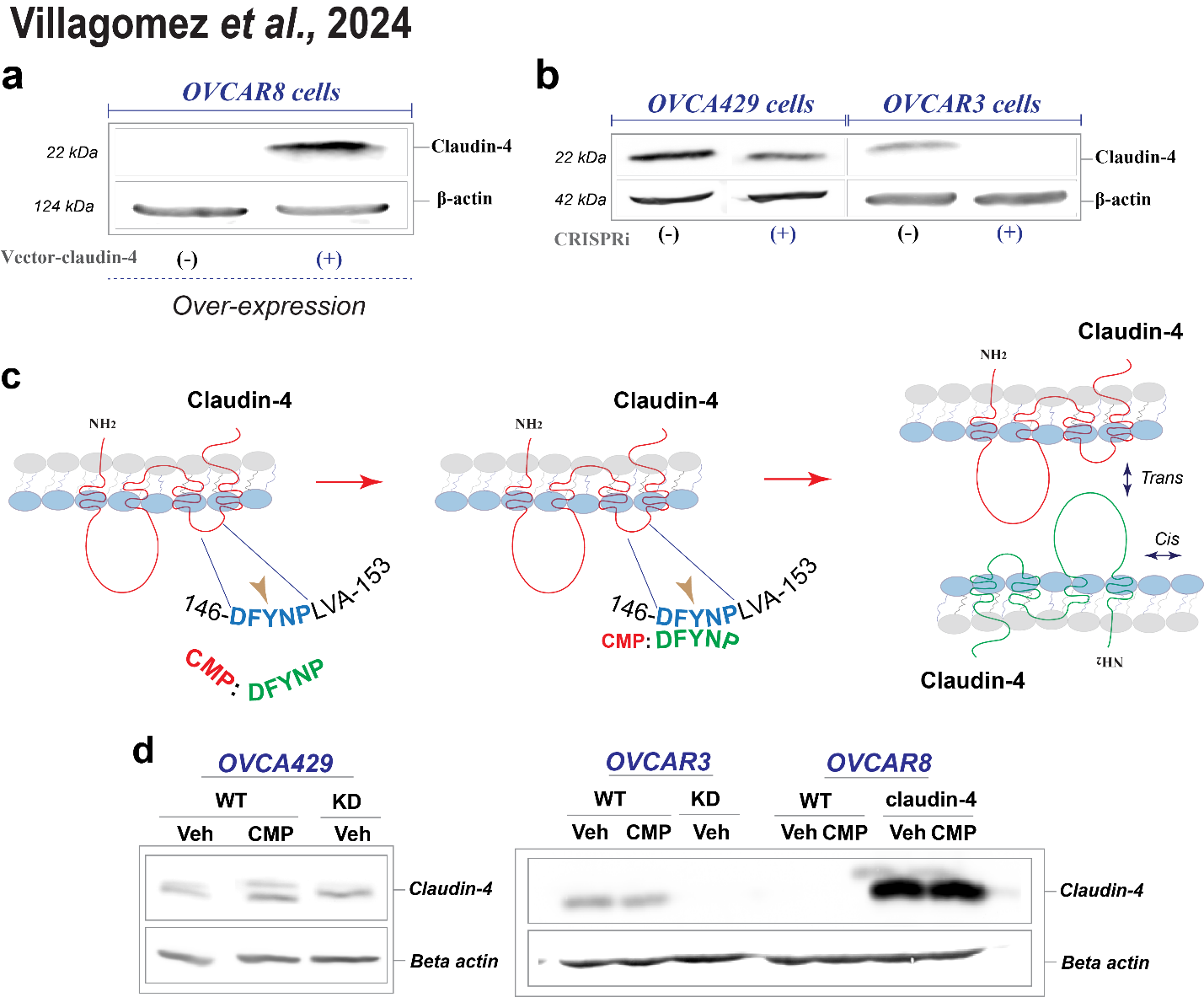


**Supplementary Figure 1. (a)** Verification of claudin-4 overexpression (left) in OVCAR8 cells (which do not express claudin-4) and its downregulation (knockdown using CRISPRi in OVCA429 and OVCAR3 cells) (**b**) by immunoblotting. **(c)** Drawing highlighting the structure of claudin-4, observing two extracellular loops (large and small). It also illustrates that CMP (claudin mimic peptide), a small peptide containing 5 amino acids (DFYNP), targets a conserved sequence in claudin-4 localized in the small extracellular loop, which potentially interferes with claudin-4 interaction with other protein partners. (1-4) (**d**) Immunoblotting for claudin-4 after treatment CMP treatment (400µmol/L (µM) for 48h), highlighting no evident changes in claudin-4 expression due to CMP treatment.
