# Supplementary material for: Claudin-4 Stabilizes the Genome via Nuclear and Cell-Cycle Remodeling to Support Ovarian Cancer Cell Survival
Source: Cancer Res Commun. 2025 Jan 7;5(1):39–53. doi: 10.1158/2767-9764.CRC-24-0558 (PMC11705808; doi:10.1158/2767-9764.CRC-24-0558)
Supplement: Supplementary Figure 2 — Claudin-4 dependent cell cycle and Lamin expression. [file crc-24-0558_supplementary_figure_2_suppsf2.docx]

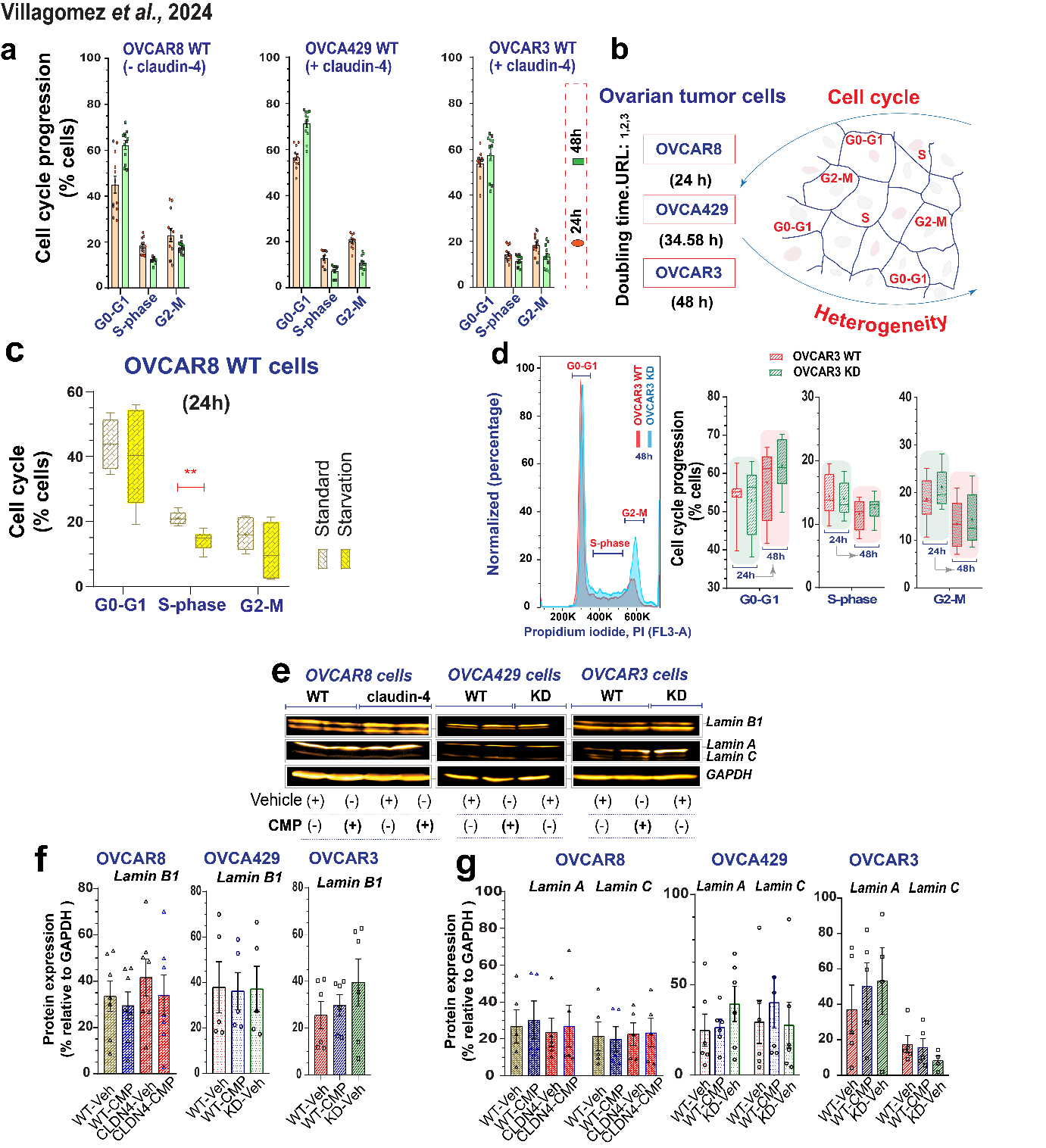
**Supplementary Figure 2.** **(a)** Graphs show WT ovarian tumor cells in different phases of the cell cycle (green symbol at 24h; red symbol at 48h). (**b**) Drawing highlighting heterogeneity of ovarian tumor cells, indicated as localization in different phases of cell cycle (references: URL 1-3). Also, it highlights differences in doubling time among different ovarian tumor cells. (**c**) Representative histograms of cell cycle phases during claudin-4 downregulation in OVCAR3 cells at 24h and 48h (4 independent experiments, Two-tailed Unpaired t test; significance p<0.05; Graphs show min to max, + indicates mean). (**d**) Quantification of cell cycle progression by flow cytometry of propidium iodide-stained OVCAR8 WT cells during standard (RPMI, 10% FBS) culture conditions or starvation (RPMI, 1% FBS). (**e**) immunoblotting for lamin B1 and lamin A/C and corresponding quantification (**f**, lamin B1; **g**, lamin A/C) relative to loading control (4 independent experiments), respectively. (One-way ANOVA and Tukey's multiple comparisons test; p<0.5). Graphs show mean and SEM.
