# Supplementary material for: Claudin-4 Stabilizes the Genome via Nuclear and Cell-Cycle Remodeling to Support Ovarian Cancer Cell Survival
Source: Cancer Res Commun. 2025 Jan 7;5(1):39–53. doi: 10.1158/2767-9764.CRC-24-0558 (PMC11705808; doi:10.1158/2767-9764.CRC-24-0558)
Supplement: Supplementary Figure 3 — Gating strategy for hypertetraploid aneuploidy in epithelial ovarian cancer cells. [file crc-24-0558_supplementary_figure_3_suppsf3.docx]

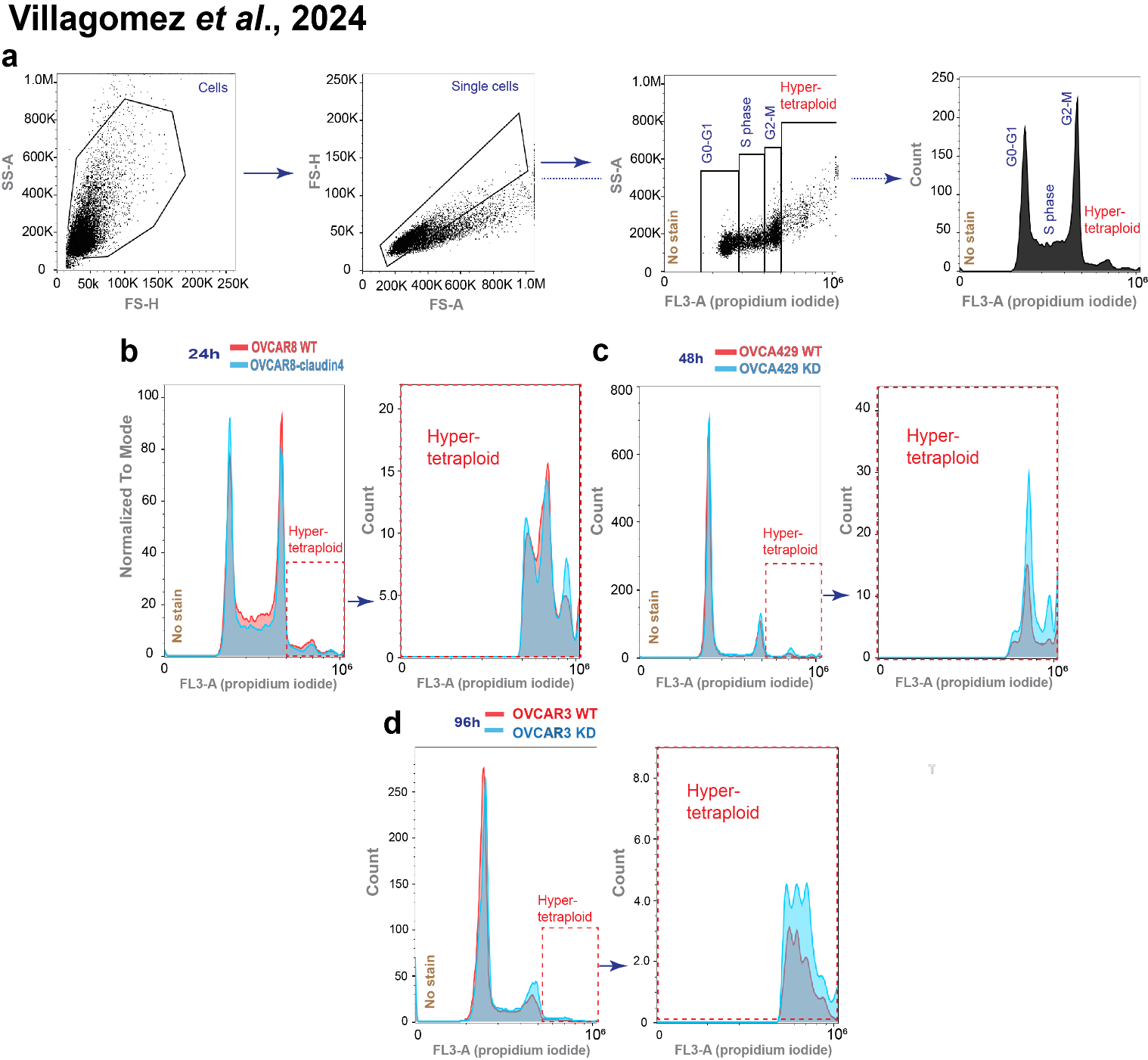


**Supplementary Figure 3.** Flow cytometry strategy to localize hypertetraploid aneuploidy in epithelial ovarian cancer cells. (**a**) Dot plots show a population of cells, then a gate for single cells, and finally a gate for propidium iodide (PI) positive cells. On the right, the same gate is shown in histogram. (**b**) Selected histogram for OVCAR8 cells positive for PI during cell cycle at 24h and gate for hypertetraploid aneuploidy. (**c**) Similar information is shown for OVCA429 at 48h and OVCAR3 cells at 96h (**d**).
