# Supplementary material for: Claudin-4 Stabilizes the Genome via Nuclear and Cell-Cycle Remodeling to Support Ovarian Cancer Cell Survival
Source: Cancer Res Commun. 2025 Jan 7;5(1):39–53. doi: 10.1158/2767-9764.CRC-24-0558 (PMC11705808; doi:10.1158/2767-9764.CRC-24-0558)
Supplement: Supplementary Figure 4 — Nuclei morphology following claudin-4 inhibition. [file crc-24-0558_supplementary_figure_4_suppsf4.docx]

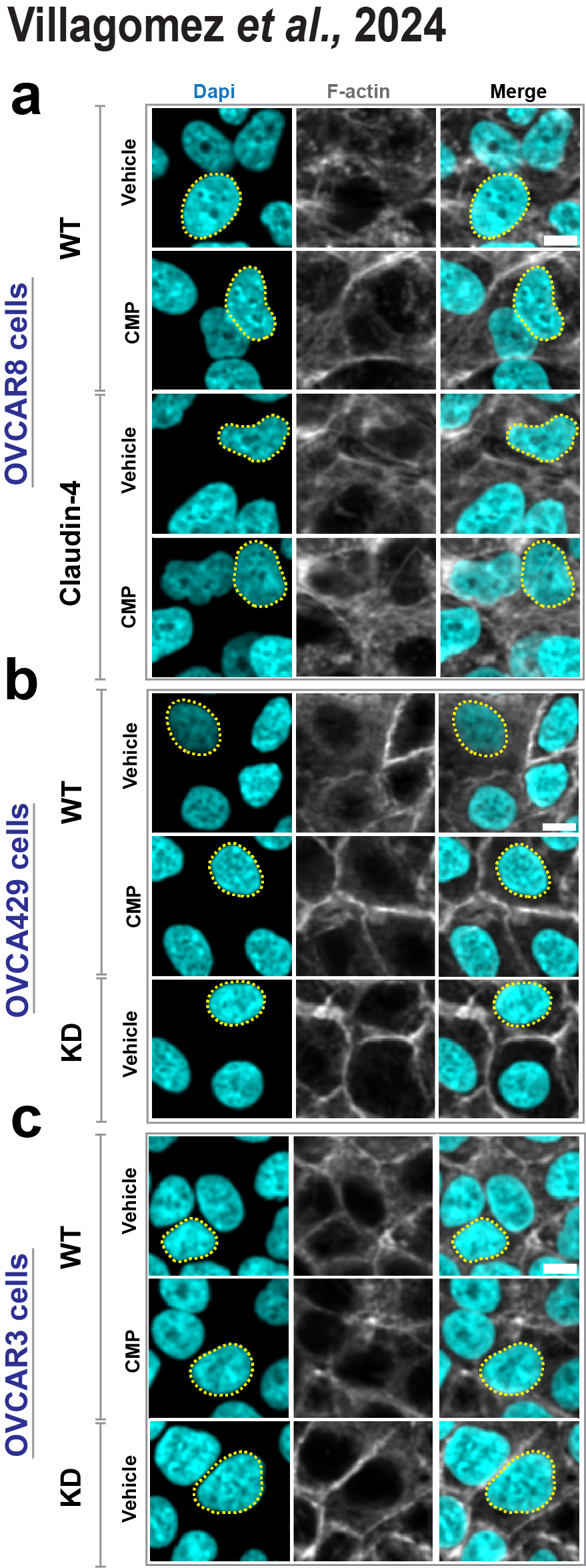


**Supplementary Figure 4.** Epithelial ovarian cancer cells were treated with CMP (400µmol/L) for 48h. Subsequently, cells were stained with dapi (nuclei) and phalloidin (F-actin) to carry out a morphometric characterization. (**a**), (**b**), and (**c**) show representative confocal images of OVCAR8, OVCA429, and OVCAR3 cells, respectively. Scale bar 10µm.
