# Supplementary material for: Claudin-4 Stabilizes the Genome via Nuclear and Cell-Cycle Remodeling to Support Ovarian Cancer Cell Survival
Source: Cancer Res Commun. 2025 Jan 7;5(1):39–53. doi: 10.1158/2767-9764.CRC-24-0558 (PMC11705808; doi:10.1158/2767-9764.CRC-24-0558)
Supplement: Supplementary Figure 5 — Claudin-4 dependent actin reorganization. [file crc-24-0558_supplementary_figure_5_suppsf5.docx]

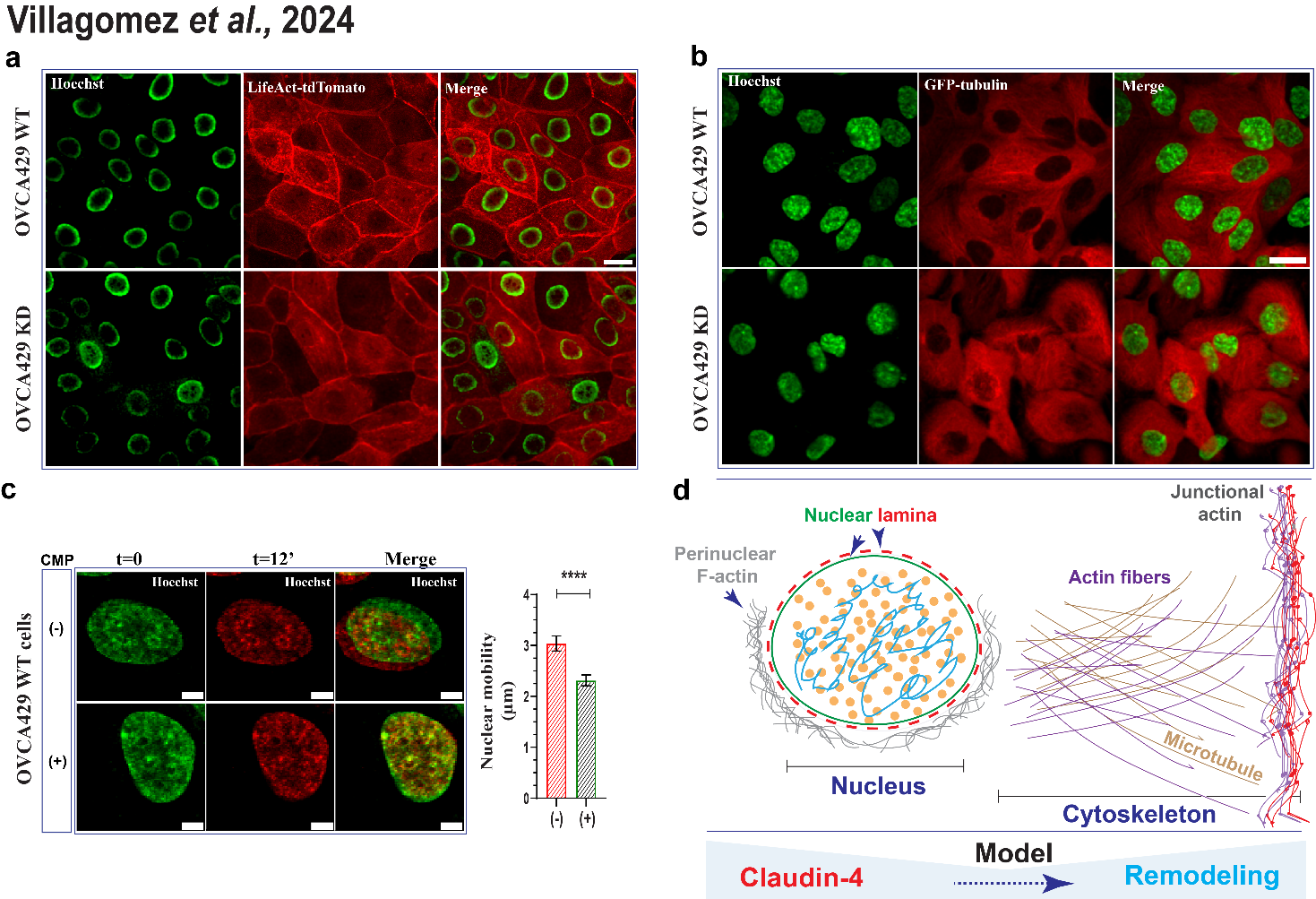


**Supplementary Figure 5.** Live cell imaging evaluation of ovarian tumor cells. (**a**) Shows representative confocal images of living cells expressing LifeAct-tdTomato (marker of F-actin) without any stimuli. (**b**) shows representative confocal images of living cells (xyt; maximum projections) of cells expressing GFP-tubulin without any stimuli. (**c**) Shows selected confocal images (xyt/30min/37ºC) of nucleus at different time points (cells treated with CMP; 400µmol/L for 24h or left untreated), which were overlaid to highlight the nuclear mobility; right, quantification of nuclear mobility (n= no treated, 44 cells; CMP treated, 41 cells; 4 independent experiments; Kruskal-Wallis test and Dunn’s multiple comparison test, p<0.5). (**d**) Presents a model that highlights our findings on the role of claudin-4 in the remodeling of the nucleus and cytoskeleton. Nuclear remodeling was linked to alterations in lamin B1 localization, resulting in changes to the nuclear lamina, along with the accumulation of perinuclear F-actin. Cytoskeletal remodeling involves changes in actin fibers, the microtubule network, and junctional F-actin, which supports cell-to-cell junctions, potentially impacting nucleus positioning, cell cycle, and cell morphology. Graph shows mean and SEM, scale bars 5µm (**c**) and 20µm (**a, b**).
