# Supplementary material for: Claudin-4 Stabilizes the Genome via Nuclear and Cell-Cycle Remodeling to Support Ovarian Cancer Cell Survival
Source: Cancer Res Commun. 2025 Jan 7;5(1):39–53. doi: 10.1158/2767-9764.CRC-24-0558 (PMC11705808; doi:10.1158/2767-9764.CRC-24-0558)
Supplement: Supplementary Figure 6 — Claudin-4 modulation and responses to forskilin and olaparib. [file crc-24-0558_supplementary_figure_6_suppsf6.docx]

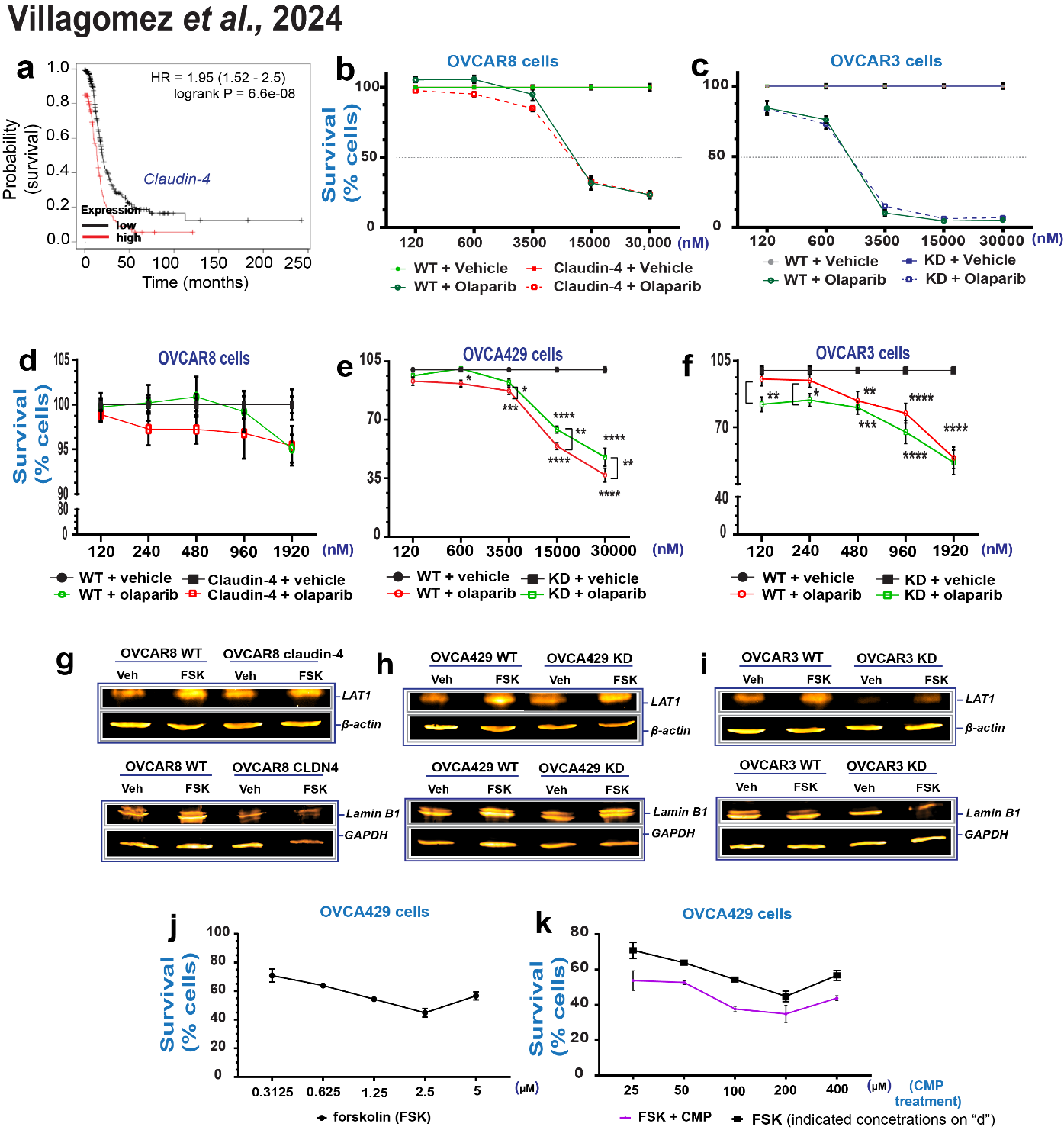
**Supplementary Figure 6.** (**a**) shows a Kaplan-Meier curve based on claudin-4 expression in human ovarian tumors (Kaplan-Meier Plotter), highlighting the association of higher claudin-4 expression with reduced patient survival. (**b**) and (**c**) show survival of ovarian tumor cells treated with varying concentrations of olaparib treatment in cells where claudin-4 was overexpressed (OVCAR8 cells) or downregulated (OVCAR3 cells) to determine proper concentrations to evaluate resistance to olaparib. (**d**) Shows percentages of cell survival during treatment with selected concentrations of olaparib for OVCAR8, (**e**) OVCA429, and (**f**) OVCAR3 cells. Immunoblotting for LAT1 and lamin B1 after forskolin treatment (5µmol/L/48h) during claudin-4 overexpression in OVCAR8 cells (**g**, and **bottom**), and similar information during claudin-4 downregulation in OVCA429 cells (**h**, and **bottom**) and OVCAR3 cells (**i**, and **bottom**). (**j**) and (**k**), show percentages of ovarian tumor cell survival during various concentrations of forskolin (FSK) and a comparison with a combination of FSK at 5µmol/L with various concentrations of CMP, respectively. Graphs show mean and SEM.
