# Supplementary material for: Claudin-4 Stabilizes the Genome via Nuclear and Cell-Cycle Remodeling to Support Ovarian Cancer Cell Survival
Source: Cancer Res Commun. 2025 Jan 7;5(1):39–53. doi: 10.1158/2767-9764.CRC-24-0558 (PMC11705808; doi:10.1158/2767-9764.CRC-24-0558)
Supplement: Supplementary Figure 7 — Lamin B1 expression and nuclei morphology after tripartite treatment. [file crc-24-0558_supplementary_figure_7_suppsf7.docx]

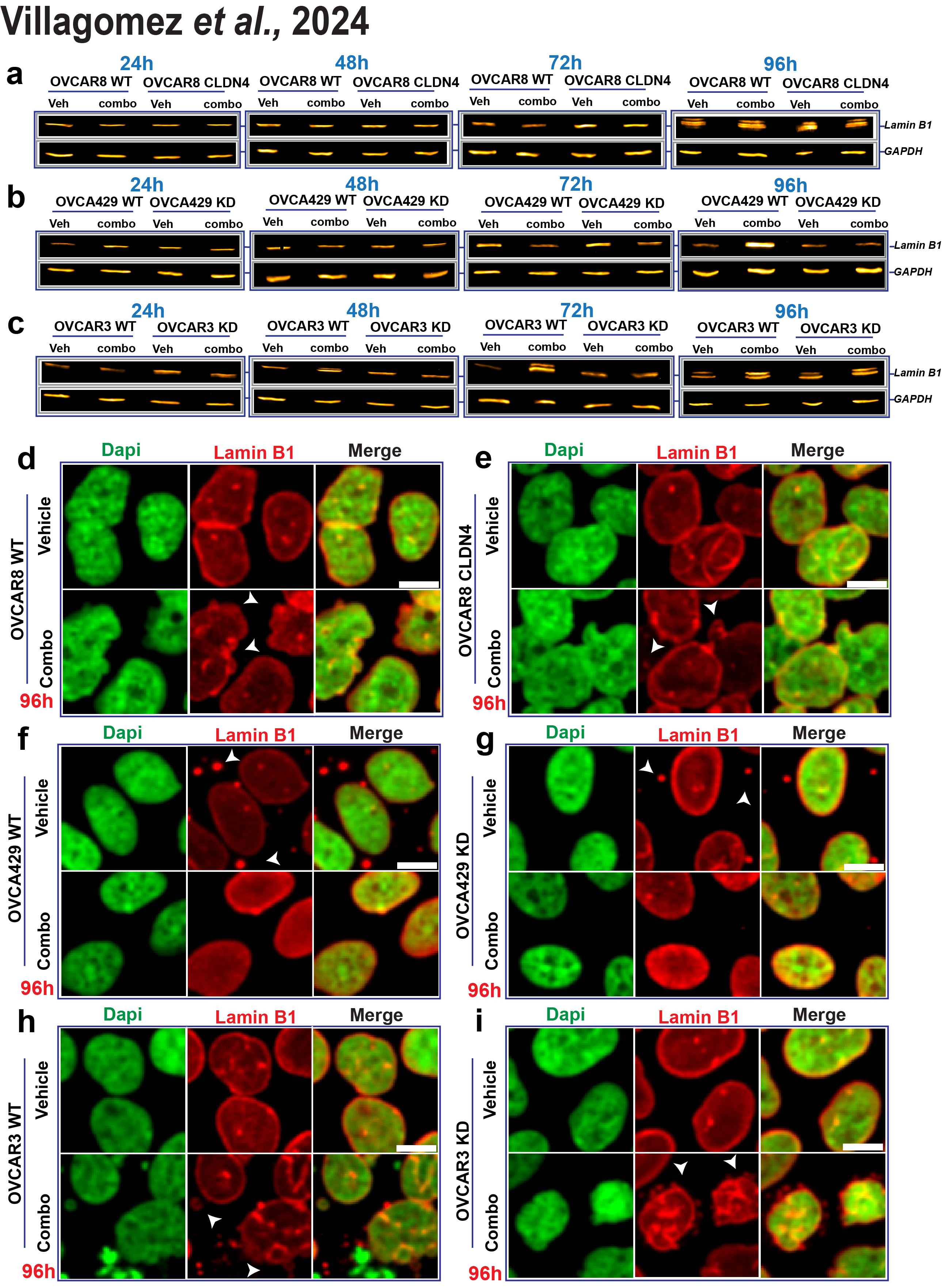
**Supplementary Figure 7.** (**a-c**) Displays lamin B1 expression via immunoblotting (IB) during tripartite treatment with olaparib (600 nmol/L), FSK (5 µmol/L), and CMP (400 µmol/L) over 24–96h in OVCAR8 (WT and claudin-4-overexpressing cells, CLDN4), OVCA429 (WT and claudin-4 knockdown cells, KD), and OVCAR3 (WT and KD) cells, respectively. Lamin B1 intracellular distribution during the same treatment at 96h is shown in OVCAR8 WT (**d**) and CLDN4 cells (**e**). Similar data are presented in (**f** and **g**) for OVCA429 WT and KD cells, and in (**h** and **i**) for OVCAR3 WT and KD cells, respectively. Scale bar: 10 µm.
