# Supplementary material for: Claudin-4 Stabilizes the Genome via Nuclear and Cell-Cycle Remodeling to Support Ovarian Cancer Cell Survival
Source: Cancer Res Commun. 2025 Jan 7;5(1):39–53. doi: 10.1158/2767-9764.CRC-24-0558 (PMC11705808; doi:10.1158/2767-9764.CRC-24-0558)
Supplement: Supplementary Figure 8 — Reactive oxygen species positive control in cell lines. [file crc-24-0558_supplementary_figure_8_suppsf8.docx]

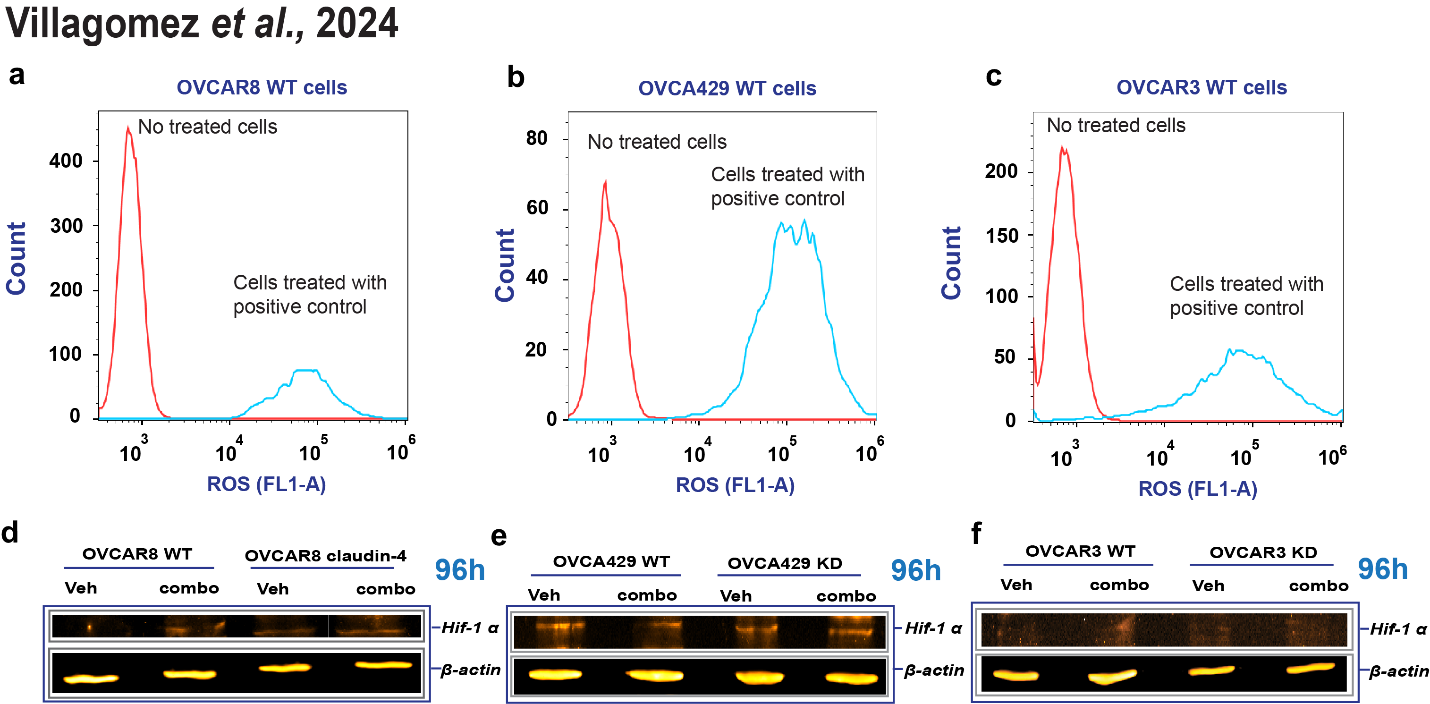


**Supplementary Figure 8.** (**a**), (**b**), and (**c**) are histograms confirming reactive oxygen species (ROS) generation by OVCAR8, OVCA429, and OVCAR3 cells, respectively, using TBHP at 250µM as a positive control. (**d**), (**e**), and (**f**) show HIF-1 alpha protein expression during tripartite combination treatment of olaparib (600nmol/L), FSK (5µmol/L), and CMP (400µmol/L) at 96h of treatment in OVCAR8, OVCA429, and OVCAR3 cells, respectively.
